# Certified Peer Support Specialists Training in Technology and Delivery of Digital Peer Support Services: Cross-sectional Study

**DOI:** 10.2196/40065

**Published:** 2022-12-07

**Authors:** Karen Fortuna, Julia Hill, Samantha Chalker, Joelle Ferron

**Affiliations:** 1 Geisel School of Medicine Department of Psychiatry Dartmouth College Concord, NH United States; 2 Dartmouth College Hanover, NH United States; 3 Veterans Affairs San Diego Healthcare System San Diego, CA United States

**Keywords:** digital peer support, mHealth, COVID-19, mental health, remote service, remote mental health, telehealth, peer support, psychological health

## Abstract

**Background:**

When the COVID-19 pandemic lockdown measures were instituted, the wide-scale necessity for remote mental health care increased among professional clinicians, such as psychiatrists, psychologists, social workers, and certified peer support (CPS) specialists. Factors contributing to increased demand include concern for the safety of loved ones, the safety of oneself, overall well-being, unemployment, and loneliness for older individuals. While demand continues to increase and a shortage of mental health professionals persists, understanding the training, technology, media, and delivery of digital peer support services can facilitate community-based support services to assist patients in coping with mental health symptoms between clinical encounters with licensed professionals. Digital peer support consists of asynchronous and synchronous, live or automated, peer support services such as applications, social media, and phone calls.

**Objective:**

The purpose of this cross-sectional study is to determine how digital peer support is delivered, by which technologies it is delivered, and how certified digital peer supporters are trained within the United States to inform future delivery of digital peer support.

**Methods:**

We used an online cross-sectional self-report survey developed alongside certified peer specialists. The study included questions regarding the types of peer support training and the delivery methods used within their practices. We advertised the survey through a certified peer support specialist listserve, Facebook, and Twitter.

**Results:**

Certified peer specialists provide mutual social emotional support to those with a similar mental health condition. Of certified peer specialists trained in CPS, the majority of CPS specialists were trained in peer support (418/426, 98.1%). Peer support specialists deliver services via telephone calls (182/293, 62.1%), via videoconference-based services (160/293, 54.6%), via SMS text messages (123/293, 42%), via smartphone apps (68/293, 23.2%), and via social media (65/293, 22.2%). Certified peer specialists deliver services through virtual reality (11/293, 3.8%) and through video games (6/293, 2%). Virtual reality and video games may represent emerging technologies to develop and deliver community-based support.

**Conclusions:**

This study examined the modes of digital peer support intervention as well as the training and demographic background of peer supporters. Given the demand for mental health care, digital peer support emerges as one option to increase access. These results suggest that CPS specialists commonly use SMS text messaging, phone calls, and videoconferences to engage in peer support. Less frequently, they may use diverse modes such as apps, social media, and video games. It is important to consider the backgrounds of peer supporters and the mediums of communication to best accommodate areas where access to peer support is emerging. Larger longitudinal studies and a variety of experimental designs may be considered to understand the efficacy of digital interventions and digital peer support training to direct optimal care.

## Introduction

Social distancing and lockdown measures due to the COVID-19 pandemic have transformed the delivery of mental health care from in-person to remote services supported through the use of technologies [1]. One study showed that, before the COVID-19 pandemic, 80% of mental health practitioners (ie, social workers, psychiatrists, and psychologists) did not offer remote services, while as of late March 2020, that number had declined dramatically to *only* 19% [1].

When the COVID-19 pandemic lockdown measures were instituted, a wide-scale necessity for remote mental health care emerged not only for professional clinicians, such as psychiatrists, psychologists, and social workers, but also for certified peer support (CPS) specialists [2]. CPS specialists (ie, individuals who provide mutual, social, and emotional support to those with a similar mental health condition and are trained and accredited to offer services like text-based advice or encouragement toward well-being in between clinical encounters with psychiatric treatment teams [3]) are mobilized to offer digital peer support services through technologies such as videoconferencing platforms and smartphone apps. Digital peer support has been shown to help patients in between clinical encounters and impact quality of life, functioning, and medical and mental health self-management skill development, and promote engagement with in-person and digital services [4]. Understanding the training and delivery of digital peer support services can facilitate community-based support services to assist patients in coping with mental health symptoms between clinical encounters with licensed professionals. In this cross-sectional study, we describe the training and delivery of digital peer support within the United States.

## Methods

### Development

A national online survey was developed with input from certified peer specialists: 2 CPS specialists, who were not authors of the study, and were selected from the Collaborative Design for Recovery and Health, a volunteer, virtual collaborative of service users, peer support specialists, caregivers, policy makers, and scientists with and without a lived experience engaging in community-based participatory research [5]. They reviewed and modified questions to ensure clarity and appropriateness for respondents. The requirements for authorship according to the New England Journal of Medicine are (1) substantial contributions to the conception and design or acquisition, analysis, or interpretation of data; (2) drafting of the article or critical revision for important intellectual content; (3) final approval of the version to be published; and (4) agreement to be accountable for all aspects of the work in ensuring that questions related to the accuracy or integrity of any part of the article are appropriately investigated and resolved. The certified peer specialists did not meet these requirements and were not interested in authorship.

### Delivery

Qualtrics was used to deliver the online survey. We sampled participants through an online newsletter announcement through the last author’s CPS specialist listserve. The newsletter was sent 3 times to individuals over the course of a month. The listserve includes 1500 CPS specialists. This link and scripted text announcing the study were also sent out via popular peer support Facebook media outlets and Twitter. The response rate is not known due to the unreported reach of social media. The survey was available online from September 2020 to November 2020. We included individuals who completed a state-accredited peer-support training program that resulted in certification, resided in the United States, and were older than 18 years. While there were 464 respondents to the survey, we included data from a total of 426 peer support specialists who had either fully responded to all survey questions or to the majority of questions; the 38 others with less than half to no responses were omitted from the analyses.

### Ethical Considerations

This cross-sectional study was approved by the Dartmouth-Hitchcock Health institutional review board (# STUDY02000514). A consent form was provided to participants online. Typed consent was required to proceed to complete the survey. The study presents the reporting guidelines for cross-sectional studies according to the organization STROBE (Strengthening the Reporting of Observational Studies in Epidemiology) [6].

### Measures

The survey began with questions about sociodemographic information such as “What state in the United States do you represent? [drop down select]” and “What is your race/ethnicity? [select all that apply] White, Black or African American, Native American or Alaska Native, Asian, Native Hawaiian or Pacific Islander, Hispanic.” Given their role as CPS specialists, they were also asked, “If comfortable, what type of lived experience have you been impacted by [please select the primary]? Schizophrenia, Schizoaffective Disorder, Bipolar Disorder, Major Depression, Other Mental Health Concerns, Alcohol Use Disorder, Opioid Use Disorder, Other Substance Misuse Concerns, Physical Health Concerns, None of the above.”

Next, they were asked about employment and training in peer support. For example, “Are you trained in offering peer support? [select all that apply] Yes, I am a peer support specialist (including certified and non-certified), Yes, I am a recovery coach, Yes, I am an older adult peer support specialist, Yes, I am a Veteran peer support specialist, No, Other.” and “If you selected ‘other,’ please explain [open answer].” They also answered questions about the use of technology for the delivery of peer support, for example, “If you are offering digital peer support, how are you providing service to service users? Please select all modes by which you are providing digital peer support: Smartphone application, Text messaging, Phone calls, Videoconference, Social media, Video game.” Last, we asked an open-ended question regarding how participants used technologies to deliver treatment, for example, “If you selected ‘other,’ please explain the other types of technology service users use to support their recovery: [open answer].” The online survey took approximately 10 minutes to complete.

### Analysis

We used descriptive statistics (ie, frequencies and percentages) to assess the demographic characteristics of the sample. We used listwise deletion and description to address missing data. We conducted a chi-square analysis to examine the substantial association between the mode of therapy and the lived experience of mental illness. Analyses were conducted using SPSS 17 (IBM Corp).

## Results

A total of 426 peer support specialists responded to the national online survey from 42 states as shown in Table 1. Geographic location was distributed across the United States, with the largest proportions being from Tennessee (57/426, 12.3%) and from Virginia (64/426, 13.8%). Most offered digital peer support (295/426, 71.1%), and most were trained in peer support (418/426, 98.1%). Demographics are shown in Table 2.

The technology used during digital peer support services is shown in Table 3. Most CPS specialists’ services were delivered through phone calls (182/293, 62.1%), videoconference-based services like Zoom (160/293, 54.6%), or text (123/293, 42%). Peer support specialists also used smartphone apps (68/293, 23.2%), including apps such as Calm, 12 Step Tool Kit, NA Meeting Guide, Affirmations, Celebrate Recovery, BoosterBuddy, Easyquit, Connections Companion app, DayCount, Daylio, Headspace, Mindfulness Coach, WRAP, and PeerTECH, and social media (65/293, 22.2%), including Facebook. Of the 120 smartphone apps mentioned, 4 (3.3%) targeted connection making, 5 (4.2%) targeted alcohol or cigarette use, and 6 (5%) targeted meditation and mindfulness. Some emerging digital peer support services were offered through virtual reality (11/293, 3.8%) and video games such as Words With Friends, Mah Jong, Sims, Fallout, and EVE Online (6/293, 2%).

For the section of Table 2 labeled “type of organization as paid or volunteer PSS” some examples of other types of organizations include “I am both a provider of peer support and recovery coaching as well as Trauma-Informed Peer Support” or “Certified Community Health Worker with lived experience” or “NA.”

A chi-square test for goodness of fit showed a substantial relationship between CPS use of video games for their own recovery and primary lived experience of mental health (χ^2^_8_=24.4; *P*=.002). The data indicate that the use of video games by CPS for peer support varies based on the primary type of lived experience. A chi-square test for goodness of fit showed a substantial relationship between CPS use of smartphone apps and primary lived experience of mental health (χ^2^_8_=16.4; *P*=.04) Those with opioid use disorder and other substance misuse concerns used smartphone apps less than others with different primary lived experiences. Those with depression used smartphone apps more than others with different primary types of lived experience. The data indicate that the use of smartphone apps by CPS for peer support varies based on the primary type of lived experience.

**Table 1:** Proportion of respondents from each state of the United States.

State	Respondents, n (%)
Alabama	11 (2.4)
Alaska	1 (0.2)
Arizona	6 (1.3)
Arkansas	4 (0.9)
California	2 (0.4)
Colorado	3 (0.6)
Connecticut	2 (0.4)
Florida	5 (1.1)
Georgia	2 (0.4)
Hawaii	1 (0.2)
Idaho	1 (0.2)
Illinois	21 (4.5)
Indiana	38 (8.2)
Iowa	4 (0.9)
Kansas	1 (0.2)
Kentucky	3 (0.6)
Louisiana	4 (0.9)
Maryland	13 (2.8)
Massachusetts	32 (6.9)
Michigan	7 (1.5)
Minnesota	4 (0.9)
Missouri	2 (0.4)
Montana	1 (0.2)
Nebraska	14 (3.0)
Nevada	1 (0.2)
New Hampshire	10 (2.2)
New Jersey	2 (0.4)
New York	12 (2.6)
North Carolina	2 (0.4)
Ohio	9 (1.9)
Oregon	6 (1.3)
Pennsylvania	8 (1.7)
Rhode Island	1 (0.2)
South Carolina	7 (1.5)
Tennessee	57 (12.3)
Texas	7 (1.5)
Utah	43 (9.3)
Vermont	3 (0.6)
Virginia	64 (13.8)
Washington	2 (0.4)
West Virginia	8 (1.7)
Wisconsin	2 (0.4)

**Table 2:** Respondent characteristics.

Sociodemographic characteristics		Respondents, n (%)
**Gender^a^**		
	Male	83 (31.7)
	Female	173 (66.0)
	Nonbinary	5 (1.9)
	Other	1 (0.4)
**Age^b^ (years)**		
	20-26	9 (3.2)
	27-49	144 (50.7)
	50-64	108 (38.0)
	≥65	23 (8.1)
**Race or ethnicity**		
	White	344 (80.8)
	Black or African American	53 (12.4)
	American Indian or Alaska Native	3 (0.7)
	Asian	4 (0.9)
	More than one race and Hispanic	14 (3.3)
	Hispanic only	8 (1.9)
**Highest grade in school completed**		
	Some elementary schooling	2 (0.5)
	Some high school	2 (0.5)
	Completed high school or GED^c^	41 (9.6)
	Some college	129 (30.3)
	Completed college or technical school	29 (6.8)
	Completed associate degree	46 (10.8)
	Completed bachelor’s degree	85 (20.0)
	Some graduate school	35 (8.2)
	Completed master’s degree	50 (11.7)
	Completed doctoral degree	7 (1.6)
**Employment status**		
	Full-time	312 (73.2)
	Part-time	61 (14.3)
	Volunteer	20 (4.7)
	Unemployed	15 (3.5)
	Retired	12 (2.8)
	Student	6 (1.4)
**Employment as trained PSS^d^**		
	No employment but trained in PSS (ie, unemployed, retired, student, volunteer)	32 (7.5)
	Volunteer or paid PSS	386 (90.6)
	Paid non-PSS	8 (1.9)
**Type of organization as paid or volunteer PSS**		369 (93.0)
	Peer-run organization	82 (19.2)
	Hospital	20 (4.7)
	Community mental health center	102 (23.9)
	Research organization	4 (0.9)
	Managed care organization	26 (5.4)
	Veterans Administration	6 (1.4)
	For-profit mental health center	9 (2.1)
	Behavioral health home	9 (2.1)
	Collaborative care model	14 (22.8)
	Other	97 (22.8)
**Trained in offering peer support^e^**		
	PSS (including certified and non-certified)	347 (81.8)
	Recovery coach	19 (4.5)
	Older adult PSS	21 (5.0)
	Veteran PSS	13 (3.1)
	Other	14 (3.2)
	No training	9 (2.1)
**Primary type of lived experience impacted by^f^**		
	Schizophrenia	11 (2.8)
	Schizoaffective disorder	9 (2.3)
	Bipolar disorder	81 (20.7)
	Major depression	122 (31.1)
	Other mental health concerns	53 (13.5)
	Alcohol use disorder	44 (11.2)
	Opioid use disorder	34 (8.7)
	Other substance misuse concerns	35 (8.9)
	Physical health concerns	3 (0.8)
**Offers digital peer support^g^**		
	Yes	295 (71.1)
	No (still a peer specialist)	120 (28.9)

^a^Missing 164 respondent answers.

^b^Missing 142 respondent answers.

^c^GED: General Educational Development test.

^d^PSS: peer support specialist.

^e^Missing 2 respondent answers.

^f^Missing 34 respondent answers.

^g^Missing 11 respondent answers.

**Table 3:** Digital peer support services technology modalities.

Modes used to provide trained digital peer support^a^	Respondents, n (%)
Smartphone apps	68 (23.2)
SMS text messaging	123 (42.0)
Phone calls	182 (62.1)
Videoconference	160 (54.6)
Social media	65 (22.2)
Video games	6 (2.0)
Virtual reality	11 (3.8)
Other	29 (9.9)

^a^Missing 133 respondent answers.

## Discussion

### Principal Findings

This web-based, cross-sectional, self-reported survey examined the training, technology, media, and delivery of digital peer support services. We found the majority of CPS specialists were trained in digital peer support, and the majority of services were delivered via telephone calls, videoconference-based services, and SMS text messages. In addition, certified peer specialists offered peer support through smartphones and social media, as well as virtual reality and video games. Virtual reality and video games may represent emerging technologies to develop and deliver digital support. The majority of CPS specialists use popular commercially available technologies (eg, Calm app) rather than mental health technologies developed by scientists and available through academic or medical institutions. Clinicians’ promotion of digital peer support services may support time between clinical encounters.

### Comparison With Prior Work

Prior work in the field of digital peer support has indicated the preliminary effectiveness of the use of digital peer support via telephone, videoconferencing, and tablet or smartphone apps. The most recent systematic review found 4 peer-reviewed articles presented on apps, 2 of which had in-person features augmented by smartphone apps, 3 on websites, 1 with social media, 2 on in-person augmented with SMS text messaging and fitness trackers, and 1 with a noninteractive psychosocial online program and email messaging [7]. However, the actual use of digital peer support technologies was not reported on in this review.

Further, prior work has discussed training in peer support services. For example, the benefits of training on mental health during the COVID-19 pandemic and increased agency were reported by certified peer specialists [8,9]. In addition to certification and training, nontrained peer support occurs through YouTube with a sense of belonging and support. Yet, the health benefits remain unclear, and there are concerns about the perceived trustworthiness of online peers and dramatization being rewarded with attention [10]. Evidence indicates that trained CPS specialists produce more robust outcomes than naturally occurring peer support. As such, there may be a need for training in this area.

### Limitations

The results should be interpreted with caution. First, these findings are limited by the cross-sectional nature of our data. Second, the authors are unable to verify that the respondents were CPS specialists. However, since there was no incentive for participation, the likelihood of recruiting ineligible participants is unlikely. Additionally, while participants report they are trained in peer support (418/426, 98.1%), training is a requirement to participate in the listserve; therefore, this may be an underestimation of those trained in peer support. Third, since this was an online national survey advertised through the last author’s listserve, Facebook, and Twitter, only people with internet access could complete the survey. This could potentially produce biased survey results (ie, those who have access to the internet or own and use smartphones might be more interested in a web-based survey on technology use, or those who cannot afford digital technology, but would be interested in using it, are unable to respond to the survey). We also did not clarify which CPS specialists were able to meet in person or did meet in person with peers, or whether or not they had access to the suggested digital technologies, such as videoconferencing. This may have caused the percent usage of digital peer support modalities to be lower as individuals with in-person opportunities or a lack of digital opportunities may use modes such as SMS text messaging, phone calls, or videoconferencing more infrequently.

### Conclusions

This study examines the modes of digital peer support intervention as well as the training and demographic background of peer supporters. Given the demand for mental health care, digital peer support emerges as one option to increase access [11]. These results suggest that among CPS specialists who own or have access to smartphones, tablets, or computers with the internet, 90%, according to a recent survey, commonly use SMS text messaging, phone calls, and videoconferencing to engage in peer support [12]. Less frequently, they may use diverse modes such as apps, social media, and video games. It is important to consider the background of peer supporters and the modes of communication to best accommodate areas where access to peer support is emerging. Larger longitudinal studies and a variety of experimental designs may be considered to understand the efficacy of digital interventions and digital peer support training to direct optimal care.

## Abbreviations

CPS: certified peer support
STROBE: Strengthening the Reporting of Observational Studies in Epidemiology
